# Developing African arbovirus networks and capacity strengthening in arbovirus surveillance and response: findings from a virtual workshop

**DOI:** 10.1186/s13071-023-05748-7

**Published:** 2023-04-14

**Authors:** Leo Braack, Shobiechah A. Wulandhari, Emmanuel Chanda, Florence Fouque, Corinne S. Merle, Udoka Nwangwu, Raman Velayudhan, Marietjie Venter, A. Gildas Yahouedo, Jo Lines, Poe Poe Aung, Kallista Chan, Tarakegn A. Abeku, James Tibenderana, Sian E. Clarke

**Affiliations:** 1 Malaria Consortium Asia, Bangkok, Thailand; 2 WHO Regional Office for Africa, Brazzaville, Congo; 3 WHO Special Programme for Research & Training in Tropical Diseases (TDR), Geneva, Switzerland; 4 National Arbovirus & Vectors Research Centre (NAVRC), Enugu, Nigeria; 5 Department for the Control of Neglected Tropical Diseases, WHO, Geneva, Switzerland; 6 Zoonotic Arbo- and Respiratory Virus Research Programme, Department of Medical Virology, University of Pretoria, Pretoria, South Africa; 7 Department of Disease Control, London School of Hygiene & Tropical Medicine, London, UK; 8 Malaria Consortium, London, UK

**Keywords:** Arbovirus, *Aedes*, Mosquito, Africa, Capacity strengthening, Surveillance, Research, Networks

## Abstract

This meeting report presents the key findings and discussion points of a 3-h virtual workshop, held on 21 September 2022, and organized by the “Resilience Against Future Threats through Vector Control (RAFT)” research consortium. The workshop aimed to identify priorities for advancing arbovirus research, network and capacity strengthening in Africa. Due to increasing human population growth, urbanization and global movement (trade, tourism, travel), mosquito-borne arboviral diseases, such as dengue, Chikungunya and Zika, are increasing globally in their distribution and prevalence. This report summarizes the presentations that reviewed the current status of arboviruses in Africa, including: (i) key findings from the recent WHO/Special Programme for Research & Training in Tropical Diseases (WHO/TDR) survey in 47 African countries that revealed deep and widespread shortfalls in the capacity to cope with arbovirus outbreak preparedness, surveillance and control; (ii) the value of networking in this context, with examples of African countries regarding arbovirus surveillance; and (iii) the main priorities identified by the breakout groups on “research gaps”, “networks” and “capacity strengthening”.

**Graphical abstract:**

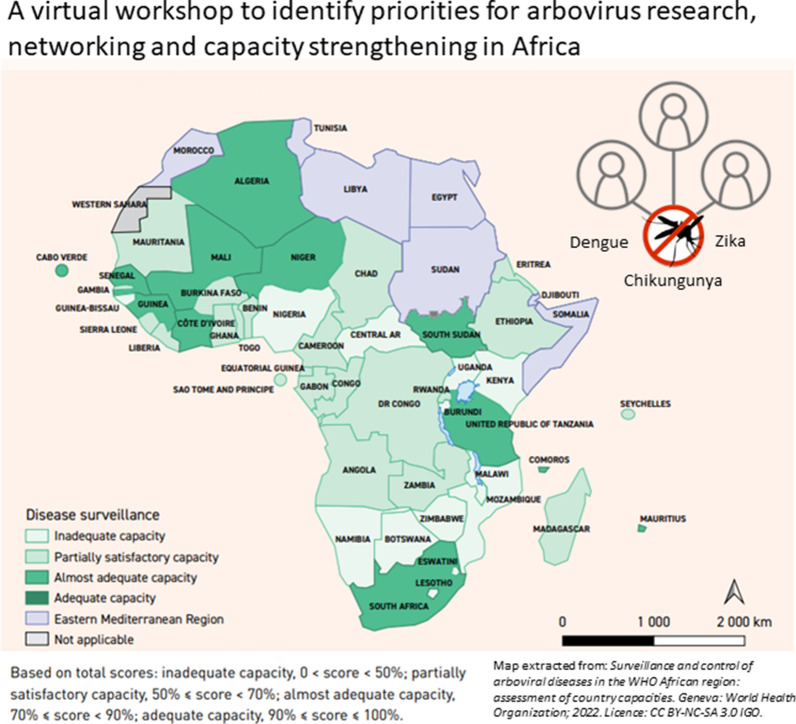

## Background

Owing to its burden, much of the world’s attention on vector-borne diseases is focused on malaria. However, the prevalence of another set of mosquito-borne diseases is increasing at a global scale, driven by anthropogenic factors, such as urbanization and globalization [1]. These diseases are caused by arboviruses (arthropod-borne viruses) and include dengue, Zika, chikungunya and yellow fever, and they have largely escaped public attention—except for the heightened media attention during the 2015–16 Zika virus epidemic in the Americas. Although mortality in infected people is lower with these diseases than with malaria, all of the aforementioned arboviruses can cause debilitating and sometimes life-threatening illnesses. For example, dengue alone results in 96 million overt cases among an estimated 390 million infected people each year and places a high economic burden on both households and governments [2–5]. Ironically, although many of these arboviruses were first identified in Africa, little is known about the prevalence and impact of these diseases on that continent. It would appear, however, that Africa is currently experiencing an increase in the spread of these viruses, and also in the spread of efficient mosquito vectors (from other continents). With growing populations, rapid urbanization, increasing global trade and movement of goods and people, Africa is a rich spawning ground for the advance of these arboviruses across the continent. Yet, historically overshadowed by malaria and vertical investments in its control, preparedness for arboviral diseases in Africa is comparatively underdeveloped.

**Fig. 1:**
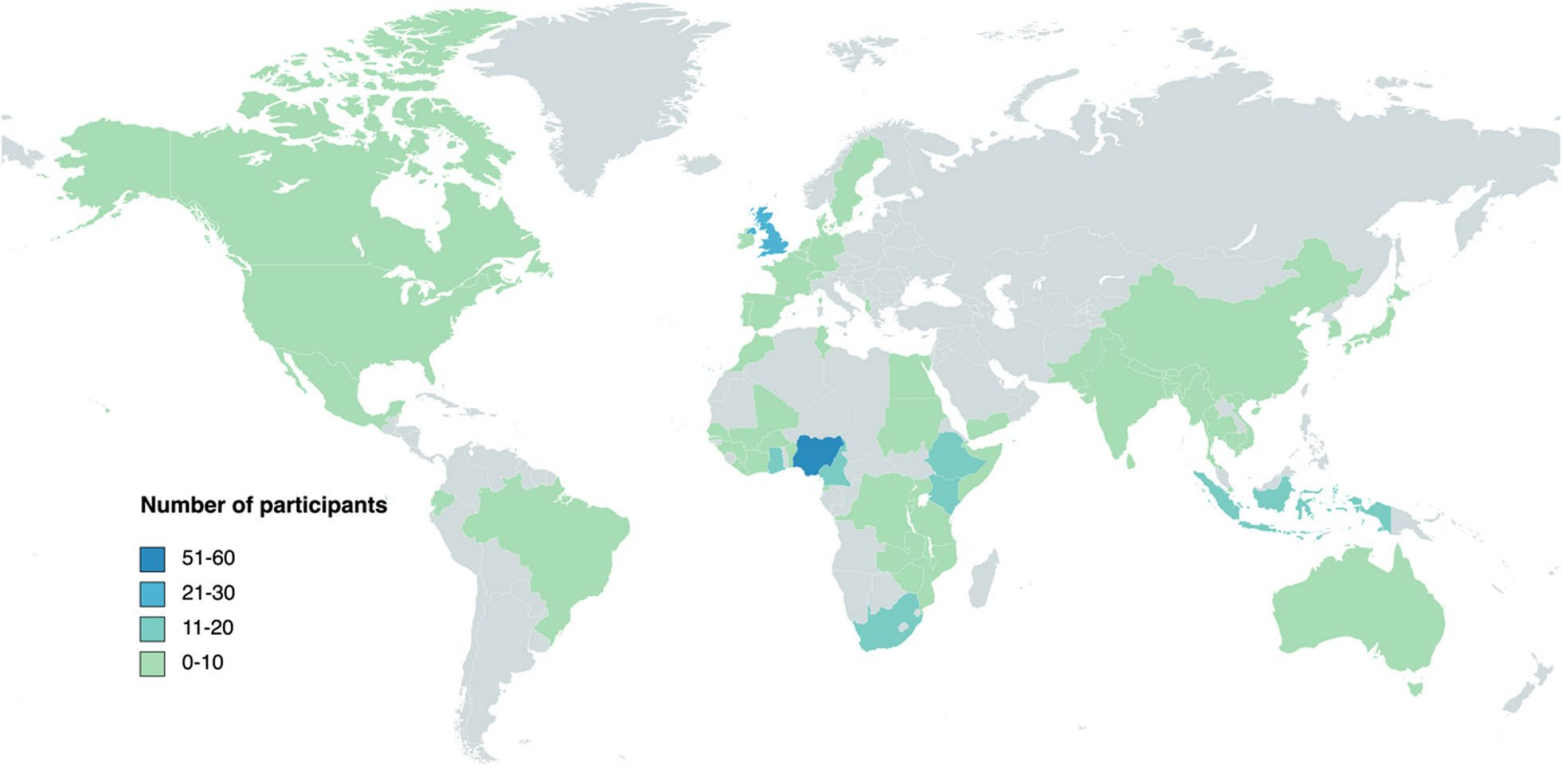
The global distribution of webinar attendees

To address this shortfall in the face of an emerging problem of arboviruses in Africa, the “Resilience Against Future Threats through Vector Control (RAFT)” research consortium held a virtual workshop on 21 September 2022. The aims of this workshop were to: (i) heighten awareness on arboviral threats among research and public health institutions in Africa; and (ii) initiate discussions on ways to strengthen institutional capacity to counter this threat. The 3-h workshop was attended by 326 participants from research/academic institutions (61%), the government sector (15%) and non-governmental organizations (NGOs, 15%). Most attendees were from Africa (63%), followed by Europe (17%) and Asia (13%) (Fig. 1).

## Virtual workshop and breakout groups

### Virtual workshop

The workshop opened with five presentations by international experts, which highlighted the following:

(i) **There is a need to monitor and control arthropod-borne viral diseases.** The WHO therefore launched the Global Arbovirus Initiative (GLAI) on 31 March 2022 to “raise the global alarm on the risk epidemics of arboviruses and potential risk of outbreaks mainly affecting urban populations” [6–8]. Raman Velayudhan (WHO/Neglected Tropical Diseases [NTD], Geneva) introduced the initiative and a list of priority actions that countries and regions can implement for increased preparedness against potential outbreaks. These priority actions (pillars) include: (i) monitoring risk and anticipate; (ii) reducing epidemic risk; (iii) strengthening vector control; (iv) preventing and preparing for pandemics; (v) enhancing innovation and new approaches; and (vi) building a coalition of partners. Further information about the GLAI can be accessed at https://www.who.int/news-room/events/detail/2022/03/31/default-calendar/global-arbovirus-initiative.
(ii) **Across Africa, the capacity to confront arboviral threats is currently inadequate. **Emmanuel Chanda (WHO Regional Office for Africa), Corinne Merle and Gildas Yahouedo (WHO/Special Programme for Research & Training in Tropical Diseases [TDR] Geneva) presented key findings from a recent WHO/TDR self-administered questionnaire in which national vector control programmes scored country and regional capacity in seven domains: (i) disease surveillance; (ii) diagnosis and case notification; (iii) management of cases and severe cases; (iv) virological surveillance; (v) routine vector surveillance and control; (vi) community sensitization and participation in non-epidemic periods; and (vii) preparedness for arboviral disease outbreaks and epidemics. Every country in the WHO Regional Office for Africa participated in the survey, and their responses revealed that the majority of the countries (41/47) and the WHO Regional Office for Africa (as a whole) had either inadequate or only partially satisfactory capacity and had sub-regional capacity of < 50%. Specific challenges identified the lack of routine entomological and epidemiological surveillance, case management guidelines, contingency plans for outbreaks/epidemics and training in the seven domains. The recommended next steps to overcome these challenges mainly involved developing training plans, regional awareness amongst health staff, advocacy campaigns for communities, funders and policymakers. A full report of the survey findings, including data for individual countries, is now available on the WHO website (https://fctc.who.int/publications/i/item/9789240052918).
(iii) **Networks are required to strengthen arboviral disease prevention and control.** Florence Fouque (WHO/TDR Geneva) highlighted the value of networks in capacity strengthening and presented examples of successful networks of relevance to arboviral threats, such as the CariVecNet (network on arboviral disease control in the Caribbean region; http://carivecnet.carpha.org/), the Worldwide Insecticide Resistance Network (WIN; https://win-network.ird.fr/) and the Global Vector Hub (https://globalvectorhub.lshtm.ac.uk/). In general, networks need to be built for stakeholders operating at all levels (from local to global) and across different disciplines (with the appropriate leadership) to aid exchanges between partners, to identify priorities, to coordinate activities and to work towards solutions.
(iv) **A One-Health strategy can be used to effectively monitor arboviruses.**
  Government agencies and arbovirus researchers can work in partnership to respond to strengthen surveillance and respond to arbovirus outbreaks. Marietjie Venter (University of Pretoria, South Africa) presented findings on the Zoonotic, Arbo- and Respiratory Virus programme (2009–2021; https://www.up.ac.za/zoonotic-arbo-and-respiratory-virus-program), which demonstrates that a comprehensive and well-integrated approach monitoring arboviral disease in sentinel animals (e.g. bird, livestock and wildlife mortalities), vectors (e.g. ticks, mosquitoes, sand flies) and humans (e.g. febrile and neurological symptoms) across multiple landscapes is highly effective in pre-emptively detecting outbreaks, reservoir hosts and vectors. Of note was the relatively high prevalence of Shuni, Sindbis, Middelburg and West Nile viruses in South Africa, as opposed to few or no cases of chikungunya and dengue, compared to other countries, which is likely due to differences in the vector species present in temperate areas [9–11]. The emergence of West Nile virus, a virus transmitted by *Culex* spp., across the Americas and Europe over the past two decades, as well as the expansion of floodwater *Aedes*-transmitted viruses and the underreporting of these viruses in Africa also suggest that these pathogens remain neglected in endemic countries and should be included in surveillance programmes.
(v) **Government agencies and arbovirus researchers can work in partnership to respond to strengthen surveillance and respond to arbovirus outbreaks.** Udoka Nwangwu (Ministry of Health [MoH], Nigeria) described the nation- and state-wide strategy against arboviral threats in Nigeria. Led by the Nigeria Centre for Disease Control (NCDC), state-wide epidemiological and entomological surveillance programmes are conducted through Emergency Operation Centres and sentinel sites set up by the National Arbovirus and Vectors Research Centre, respectively. The NCDC also leads the response to arboviral outbreaks, in collaboration with the aforementioned partners and vaccine specialists (National Primary Health Care Development Agency), state MoH and local communities. Through these approaches and in partnership with the African Centre of Excellence for Genomics of Infectious Diseases, major arboviruses (e.g. yellow fever and chikungunya) have been found to be circulating in Nigeria, where *Aedes aegypti* and *Aedes albopictus* were identified as the predominant vectors and to be spreading across urban areas [12]. Still, there are gaps in surveillance and response to outbreaks, including inadequate capacity (laboratory equipment and human expertise), training, mentorship, collaboration and funding.

### Breakout groups

Following the presentations, attendees were invited to join breakout groups to discuss one of three themes (research gaps, networking, capacity-strengthening) that can aid preparedness against arboviral threats in Africa. Guided by a series of pre-planned questions, participants in each group identified the key shortfalls, needs and priority actions for their theme (Tables 1, 2).

#### Breakout Group 1: research gaps and knowledge needs (76 participants: 83% were from research institutions, 10% from NGOs, 5% from government agencies and 2% from UN/WHO)

Improved understanding and monitoring of arbovirus epidemiology, transmission and vectors were ranked among the most important research gaps to fill (Table 1). To begin to address these gaps, collaboration with global and regional networks (Box 1) and other several follow-up actions were suggested (Table 2).

**Table 1:** Top priorities (ranked from highest to lowest) in research, networking and capacity strengthening for arboviral threats in Africa

Rank	Research/knowledge gaps^a^	Networking	Capacity strengthening
1	Improved understanding of arbovirus epidemiology (incl. transmission patterns, disease trends and disease burden)	Modelling	Skills in pathogen detection – specifically, laboratory capacity in molecular identification and bioinformatics
2	Routine surveillance of arbovirus diversity, prevalence and vectors	Diagnostics, case management and serum screening	Skills in vector surveillance
3	More precise understanding of vector species involved in transmission	Outbreak management	Improve access to training materials
4		Molecular (including general laboratory) techniques and investigations	Skills in arbovirus case management
5		Vector surveillance, including mosquito infection prevalence	Skills in disease modelling and prediction
6		Capacity strengthening, training and job opportunities	
7		Linking across language barriers in Africa	
8		Data sharing across countries, especially on disease prevalence and status	
9		Links between government, private sector and communities	
10		Community engagement	

^a^Note that priorities originally suggested (by respondents) were re-grouped to ensure that they complement each other

**Table 2:** Top three immediate next steps identified to support progress in research, networking and capacity strengthening for arboviral threats in Africa

Next steps	Research/knowledge gaps	Networking	Capacity strengthening
1	Questionnaire survey to compile a more in-depth list (and ranking) of research priorities	Report on and distribute findings of the current workshop (Sept 2022)	Report on and distribute findings of current workshop breakout groups (Sept 2022)
2	Questionnaire survey to identify institutions involved in arbovirus research in Africa and collect more detailed information on the nature of their research, technical capacity, and areas of expertise	Follow-up with structured online questionnaire survey to unpack and prioritize needs for networking	Follow-up with structured online questionnaires to develop a more granular understanding of specific country needs and priorities [which is now provided in the WHO/TDR survey]
3	Hold another follow-up webinar to discuss key knowledge gaps identified in the above survey and discuss how research institutions can address the required research	Hold another webinar (with breakout groups) to present and discuss findings from the above survey	Hold another webinar (with breakout groups) to present and discuss findings from the above survey

*TDR* WHO/Special Programme for Research & Training in Tropical Diseases

Box 1. Networks already existing that provide some support for arbovirus issues, identified by breakout group participants.

1. Africa Centres for Disease Control and Prevention (Africa CDC)
2. African Gene Drive for Vector Control (AGDVC) network
3. Global Vector Hub
4. Pan-African Mosquito Control Association (PAMCA)
5. Partnership for Increasing the Impact of Vector Control (PIIVeC)
6. Prepare4VBD
7. Southern African Centre for Infectious Disease Surveillance (SACIDS)
8. West African Aedes Surveillance Network (WAASuN),
9. Worldwide Insecticide resistance Network (WIN)
10. Pasteur Institut network

#### Breakout Group 2: networking needs (45 participants: 71% from research institutions, 21% from regional level and UN agencies and 8% from NGOs)

A total of 10 networks (4 global and 6 regional) that currently provide some level of support on arbovirus issues were identified (Box 1). Nonetheless, despite this proliferation of networks, participants identified several unmet needs. Requirements in networking that were not already covered by the named, existing networks are listed in Table 1. Respondents also indicated that funding was required to establish these networks, followed by a database of specialists and training opportunities. The group also defined the core objectives and activities that should be captured in an African research network for arboviruses (Box 2).

Box 2. Core objectives and activities of an African arbovirus network^1^

1. Evaluate burden of arbovirus across Africa,
2. Coordinate surveillance practices between countries,
3. Monitor outbreak spread,
4. Connect laboratories to establish common Standard Operating Procedures,
5. Capacity strengthening,
6. Coordinating diagnostic, surveillance and control procedures,
7. Promote collaborative research,
8. Platform for workshops and seminars, and
9. Data-sharing.

#### Breakout Group 3: capacity-Strengthening Needs (52 participants: 65% from research institutions, 19% from NGOs, 10% from UN/WHO and 6% from government agencies)

Respondents listed the top three capacity-strengthening needs most relevant to their countries and then ranked them according to priority (Table 1). Among these, the most urgent priority was training in vector surveillance and pathogen detection. However, with few participants from MoH departments of the various governments, which are generally responsible for arbovirus surveillance and response, the validity of responses from this group may be limited. A follow-up survey directed to government agencies may be required. When asked to identify institutions or sources of expertise in Africa that could provide relevant training, a total of 10 institutions^2^ located in five countries were listed. The distribution of expertise needs to be better distributed geographically across East (*n* = 2 institutions), West/Central (*n* = 8) and South (*n* = 0) Africa.

Finally, participants in each breakout group were asked to vote/rank a set of potential immediate follow-up actions to help support forward progress under each theme (Table 2). In a post-workshop evaluation form, a majority of participants (86%) were in favour of holding regular arbovirus information-sharing webinars, at quarterly intervals, demonstrating an urgent need for more interaction and learning opportunities available upon request.

In line with the Next Steps identified by webinar participants and listed in Table 2 above, the RAFT project has now brain-stormed and outlined a series of follow-up webinars to build on the foundation provided by this first exploratory webinar. The aim is to contribute not only to an increased understanding of arbovirus issues in Africa, but also create capacity for research institutions and implementation agencies to embrace this greater awareness of needs and opportunities to undertake prioritized research, surveillance, development of national and regional guidelines, establish collaborative networks for sharing information and responding to regional needs, and share expertise. Ultimately, each country should have a National Arbovirus Control Guidelines and Action Plan, supported by an appropriate policy framework, with the capacity to implement such a Plan. Subsequent to this webinar, the RAFT project has also initiated a series of South-South Exchange Visits to bring senior managers of vector-borne disease control agencies and also leading academics from African countries to meet colleagues in some Asian and South American countries where relevant policies, systems and procedures to confront arboviral disease are more advanced, with the overall aim to learn from the experience gained by these countries.

## Conclusions

In conclusion, arboviruses are an emerging threat to Africa, but as the recent WHO/TDR survey and group discussions during this webinar demonstrate, the continent (at all levels) lacks the capacity to handle them. Not only is more research required to improve the understanding of arboviral vectors, transmission and epidemiology, but more networks (that involve training and data sharing) also need to be built to strengthen the capacity. Lessons can be learnt from the South African and Nigerian approaches to arbovirus surveillance and response.

## Data Availability

The datasets used and/or analysed during the current study are available from the corresponding author upon reasonable request.

## Abbreviations

MoH: Ministry of Health
NCDC: Nigeria Centre for Disease Control
NGOs: Non-Governmental Organizations
RAFT: Resilience Against Future Threats through vector control
WHO/TDR: WHO Special Programme for Research & Training in Tropical Diseases

## Footnotes

1 Note that these responses were extracted directly from the break-out group. Thus, further detail about specific types or areas of burden, capacity strengthening or data-sharing was not provided. It is hoped that future surveys will provide this information.

2 Available upon request.
